# Older Adults Place Greater Importance on a Purposeful Retirement

**DOI:** 10.1093/geroni/igab046.3419

**Published:** 2021-12-17

**Authors:** Rachel Best, Gabrielle Pfund, M Teresa Cardador, Victor Strecher, Patrick Hill

**Affiliations:** 1 Yeshiva University, Bronx, New York, United States; 2 Washington University in St. Louis, St. Louis, Missouri, United States; 3 University of Illinois at Urbana-Champaign, Champaign, Illinois, United States; 4 University of Michigan, Ann Arbor, Michigan, United States

## Abstract

Sense of purpose is associated with desirable health and well-being measures in older adults. Unfortunately, existing research points to complexity in the connection between purpose and retirement: some but not all people decline in sense of purpose following retirement, and some view it as nonessential to maintain a purpose specifically during retirement. These findings suggest there may be individual differences both in the importance placed on being purposeful specifically during retirement, and that there may be a discrepancy in purpose importance before retirement and during retirement. In this study, we examined whether perceived purpose importance correlates with age and personality, as well as working status. Data were collected from a U.S sample (N = 2,009), aged18-93 (M =48.51). Participants completed a survey assessing the Big Five personality traits and were asked to rate the importance of purpose before and after retirement. Findings suggest that, overall, people believe it wasrbe important to have a purpose and direction during retirement (M = 3.86). Perceived purpose importance during retirement was greater among older, conscientious, and less neurotic adults, but working status did not appear to play a role. Moreover, when comparing perceptions of purpose importance before and during retirement, age was the distinguishing factor differentiating who perceives during-retirement purpose as more important than before-retirement purpose, such that older adults placed greater importance on sense of purpose during retirement. Results indicate that older adults do value having a purpose during retirement, suggesting that purpose-focused interventions may be well-received by this population.

